# *Parenclitic* networks: uncovering new functions in biological data

**DOI:** 10.1038/srep05112

**Published:** 2014-05-29

**Authors:** Massimiliano Zanin, Joaquín Medina Alcazar, Jesus Vicente Carbajosa, Marcela Gomez Paez, David Papo, Pedro Sousa, Ernestina Menasalvas, Stefano Boccaletti

**Affiliations:** 1 Faculdade de Ciências e Tecnologia, Departamento de Engenharia Electrotécnica, Universidade Nova de Lisboa, Lisboa, Portugal; 2 Innaxis Foundation & Research Institute, José Ortega y Gasset 20, 28006, Madrid, Spain; 3 Centro de Biotecnología y Genómica de Plantas, Universidad Politécnica de Madrid, 28223 Pozuelo de Alarcón, Madrid, Spain; 4 Center for Biomedical Technology, Universidad Politécnica de Madrid, 28223 Pozuelo de Alarcón, Madrid, Spain; 5 CNR- Institute of Complex Systems, Via Madonna del Piano 10, 50019 Sesto Fiorentino, Florence, Italy

## Abstract

We introduce a novel method to represent time independent, scalar data sets as complex networks. We apply our method to investigate gene expression in the response to osmotic stress of *Arabidopsis thaliana*. In the proposed network representation, the most important genes for the plant response turn out to be the nodes with highest centrality in appropriately reconstructed networks. We also performed a target experiment, in which the predicted genes were artificially induced one by one, and the growth of the corresponding phenotypes compared to that of the wild-type. The joint application of the network reconstruction method and of the in vivo experiments allowed identifying 15 previously unknown key genes, and provided models of their mutual relationships. This novel representation extends the use of graph theory to data sets hitherto considered outside of the realm of its application, vastly simplifying the characterization of their underlying structure.

Of the different ways of representing a multi-unit system, the one afforded by complex networks^1^,^2^,^12^,^13^ is one of the most elegant and general. Often, however, defining what can indeed be treated as a system may be highly non trivial. Suppose for instance that what one wants to study is a set of biomedical data from different individuals, *e.g.* various blood tests, which are in essence but a collection of scalar values without any history. Whether and how such a matter should be treated as a unitary system is not obvious. In particular, how is one to establish which entities are inside the system and which are outside its boundaries? What would the elements be of such a system and how would internal relationships among them be defined?

*Prima facie*, such an object study would seem to lack the physical or virtual relationships between elements of the system, which anatomic brain fibres or hyper-links respectively provide for brain tissue and pages of a web site^14^. Nor would it appear to be possible to construct the sort of functional links that one can define when time evolving variables are associated to each node, as *e.g.* the time evolution of a stock price, or of brain activity in a given region^3^,^4^,^5^.

Here, we introduce a novel way of representing as networked systems such collections of isolated, possibly heterogeneous, scalars. The final result is the creation of a network for each subject, where nodes represent features, and links are weighted according to the deviation between the values of two features and their corresponding typical relationship within a studied population. The result is what we term a *parenclitic* network representation, from 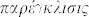, the Greek term for “deviation”, originally used by the Greek philosopher Epicurus to designate the spontaneous and unpredictable swerving of free-falling atoms, which allows them to collide^15^. Such a representation allows defining a system the identity of which parts and relationships (as well as the system's boundaries) are continuously “deviated” in a context dependent manner.

The method exploits information of a set of pre-labeled subjects to unveil the presence of reference relationships between nodes. The starting point is a multi-feature description of subjects, *e.g.* a collection of medical measurements or gene expression levels, and their affiliation to one or multiple predefined groups. While it may be unfeasible to work with the complete data set, we consider the projection of the data into all possible planes created by pairs of features. In these planes, different methods (from simple linear correlations, up to more sophisticated data mining techniques) are used to extract a reference model for each group, accounting for the characteristics of subjects. When a new, unlabeled, subject is considered, the deviation between the associated data and such reference models is used to weight the link between the corresponding nodes. See Figure 1 and Methods for a more detailed description of the whole procedure.

The reader should notice that while the reconstruction method proposed here is based on the network representation technique introduced in Ref. 16, its scope has been largely widened. While the original technique only focused on linear relations between genetic expression levels, here we introduce a general mathematical framework that is compatible with any type of relationship and any data set, as long as features (*i.e.* observables) are represented by numbers.

The topological characteristics of the resulting network can then be used to extract important information about the system. In particular, atypical conditions correspond to strongly heterogeneous networks, whereas typical or normative conditions are characterized by sparsely connected networks with homogeneous nodes^16^. Insofar as a network representation of each instance is constructed with reference to the population to which it is compared, this technique is by its very nature a difference seeker.

We present the results of the application of the parenclitic network representation to *(i)* a synthetic data set and *(ii) Arabidopsis thaliana* gene expression data. Of the wide range of transcriptomic analyses that have been performed in Arabidopsis, we selected a subset aimed at the characterization of gene expression responses under osmotic stress conditions. We illustrate the relevance of the proposed approach in the identication of key functional elements in gene reprogramming, and discuss how our methods compares with standard alternative methods.

## Results

As a first step in the test of the parenclitic method's reliability, we analyze a synthetic data set that comprises 20 instances (corresponding to sets of expression levels) and 10 features - see Methods for further details. Figure 2 reports the results obtained with this synthetic data set. The left graph depicts the behavior of features 1 and 5, for the 9 normal instances (black squares) and the abnormal one (red circle). Due to the modification of feature 5 for instance 10, the red circle deviates from the expected normal behavior (blue dashed line). The two networks, on the central and right part of the Figure, respectively represent the result of the parenclitic reconstruction technique for instances 1 and 10, *i.e.* for a normal and the abnormal one. Two important facts have to be highlighted. First, the network corresponding to the normal instance has a much lower link density than the abnormal one, correctly indicating that most pairwise gene relationships are close to the model prediction. Furthermore, the most central elements in the abnormal network are nodes 5 and 10, highlighting the two features that have been altered. It is worth noticing that other nodes may also have a central position, *e.g.* node 4, due to the noise term ζ included in the data set. Overall this result indicates that the parenclitic method correctly identifies both discordance nodes.

As a second step, we used parenclitic networks to analyze gene expression of the plant *Arabidopsis thaliana* under osmotic stress, with the objective of identifying those genes orchestrating the plant response under this specific condition. This is of particular relevance, as abiotic stresses represent the primary cause of crop loss worldwide, lowering by more than 50% the average yields of many crop plants. Therefore, a better understanding of the mechanisms behind plant responses to such stresses, starting from the genetic level, is essential.

Expression levels have been obtained from the *AtGenExpress project*^17^, including information about the 1701 genes encompassing the transcription factor repertoire^18^ represented in the Arabidopsis ATH1 array used in the study at six different time points (30 min, 1 h, 3 h, 6 h, 12 h and 24 h after stress onset).

Similar data sets have been studied in the last decade by means of different techniques, *e.g.* co-expression networks^6^,^7^,^8^ and differential-expression analysis^9^,^10^,^11^,^17^. Yet, we expect the parenclitic network approach to yield complementary results. Specifically, differential-expression analyses only focus on the evolution of expression levels through time, considering each gene as independent from the others. Co-expression networks analyze similarities between the evolutions of pairs of expression levels. Finally, the parenclitic network representation focuses on pairs of genes whose expressions depart from a reference model, thus it concentrates on differences. Furthermore, in marked contrast with classical approaches where a single network is obtained reflecting similarities across stages, in the parenclitic representation the construction of a different network for each time step allows tracking the plant response through time.

An example of the obtained networks is shown in Fig. 3 (see Methods for the details of the parenclitic representation). Namely, Fig. 3 (a) depicts the giant component of the network corresponding to 3 h after osmotic treatment. The color of links accounts for their weights, with green (red) shades indicating low (high) Z-Scores, and the size of nodes is proportional to their *α* – *centrality*^19^ - see Methods for more details. The resulting network topologies are characterized by a highly heterogeneous structure, dominated by a small number of *hubs* - as can be appreciated from the zoom reported in Fig. 3 (b). Such nodes with high centrality indicate that, at 3 h., the expression levels of the corresponding genes strongly deviate from the relationships generally established at other times. This suggests that hubs are performing some specific task at this time point, and therefore that they are key actors in regulating the overall plant response to that particular stress. The parenclitic network representation allowed identifying novel candidate genes, the full list of which is reported in Table 1, that were either previously unknown or were not considered to be related to the response to osmotic stress.

To confirm these predictions, we performed an *in vivo* screening, in which genes corresponding to the most central nodes of each graph were artificially induced in transgenic plants, and the derived phenotype after a stress response was monitored in a typical essay by measuring the length of the root of each plant - see Methods for more details on the performed experiments. As an example, Fig. 4 reports the results obtained with seven transgenic lines, *i.e.* seven groups of plants in which the expression of one gene, corresponding to a parenclitic hub, was artificially induced. Specifically, Fig. 4 (a) reports the mean length of roots for the seven lines, as compared to the normal root length in the wild type (black column) grown under osmotic stress conditions. The figure clearly visualizes the fact that, in all the seven examples, induction of the corresponding gene leads to a significant functional responses in the development of the plant. The results of the *in vivo* screening are summarized in Fig. 5. For each of the six networks analyzed, only the 20 most central genes at each time step were considered. This figure reports the number of genes already known to be relevant for the osmotic response of the plant, and the number of previously unknown genes disclosed by the parenclitic representation that have been successfully confirmed. Thus, the use of parenclitic network representations allowed the prediction -and further experimental confirmation- of key transcription factors that were not detected using alternative methodologies.

## Discussion

In conclusion, the parenclitic approach affords a network representation of data sets lacking both physical connections, and a time-varying nature. By exploiting the data associated to a set of pre-labeled subjects, and by extracting a set of reference models, it is possible to construct networks whose links represent the presence of deviations from expected relationships. The proposed methodology allowed identifying key genes regulating the response to osmotic stress of the plant *Arabidopsis thaliana*, whose role was previously unknown in the literature. The parenclitic approach also allows merging different data sources, *e.g.* gene expression levels and blood tests, into a single network portrait. Thus, our method generalizes the network representation to a very vast number of contexts and data sets that were previously thought to be outside graph theory's domain of application.

## Methods

### Parenclitic networks

Consider a set of *n* systems {*s*_1_, *s*_2_, …, *s_n_*}, each one associated to one of *n_c_* pre-defined classes - the class of each system will be denoted by 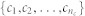. These classes represent the division of systems into different groups according to a categorical observable, whose study is the final aim of the parenclitic network reconstruction. For instance, a common biomedical problem is the classification of people as *healthy* (or *control*) or suffering from some disease; but classes may also represent people behavior under different tasks, as usually performed in the analysis of brain dynamics, or, as in the case at hand, plants under different external stresses. Each system *i* is identified by a vector of *n_f_* features 
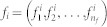
, so that each system is represented by a point in a *n_f_*-dimensional space.

The fundamental ansatz behind the proposed method is that each class can be associated to a constraint in the feature space. In other words, we suppose that a relationship 
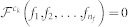
 defines the feature combination associated to the *k*-th class. In the most general case, there will be *n_c_* different relationships like 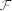, one for each of the *n_c_* classes; yet, the exact expressions of the functions 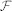 may not accessible, either because extracting them may be too complex (it may require a too high computational cost), or because not enough data are available.

**Figure 1:**
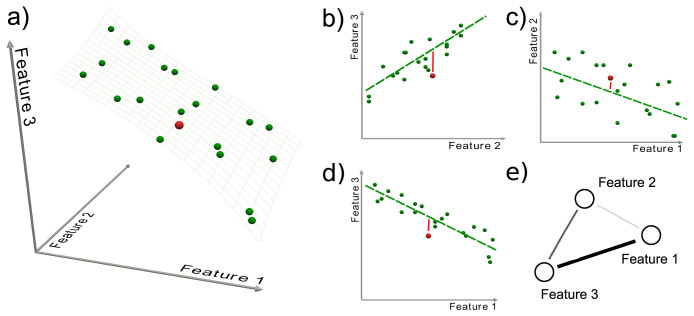
Schematic representation of the parenclitic network reconstruction method. (a) Graphical represetation of the initial data set, composed of 20 instances (systems) and three features. Each instance is represented by a green sphere, located according to the value of its features in a 3-dimensional space. The constraint surface (gray wired surface) represents the overall standard relationship 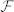 of the class. A generic unlabeled system is represented by a red sphere. (b–d) Data are then projected on each of the three possible planes. The green dashed lines represent the models extracted in each plane, *i.e.* 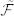. The red points are the positions of the unlabeled system, and the red lines indicate the distance of the system from the models. (e) The resulting parenclitic representation is a network where nodes are associated to features, and links are weighted according to the calculated distances (coded, in this Figure, into different line widths).

**Figure 2:**
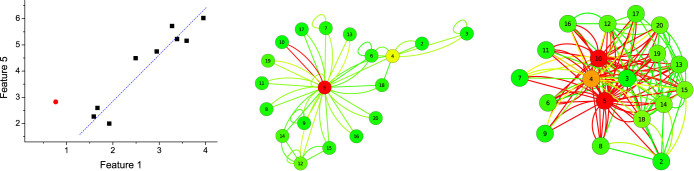
Parenclitic network reconstruction of a synthetic data set. The left graph depicts the lineal fit of features 1 and 5; black squares represent instances 1–9, while the red dot represents instance 10 (the one with abnormal expression levels). The central (right) network is the result of the parenclitic reconstruction process for instance number 5 (10), *i.e.* for a normal (abnormal) instance. Nodes and links are coloured respectively according to their *α*-centrality and weight, from green (low) to red (high). For the sake of clarity, only links with weight greater than 1.5, and nodes connected to the giant component of the network are represented.

**Figure 3:**
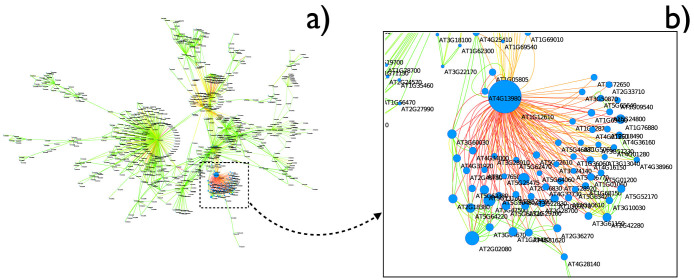
Parenclitic network for the response of Arabidopsis thaliana to osmotic stress after 3 h. (a) Representation of the giant component of the network; for the sake of clarity, links with weight lower than 3 are not depicted. (b) Magnification of the neighborhood of the most central node, *AT1G12610*. Notice that labels are positioned in the lower right corner of each node - thus *AT4G13980* is the label of the small one one the left. In both cases, color represents the link weight (from green to red), and node size is associated with the corresponding value of *α*-centrality.

**Figure 4:**
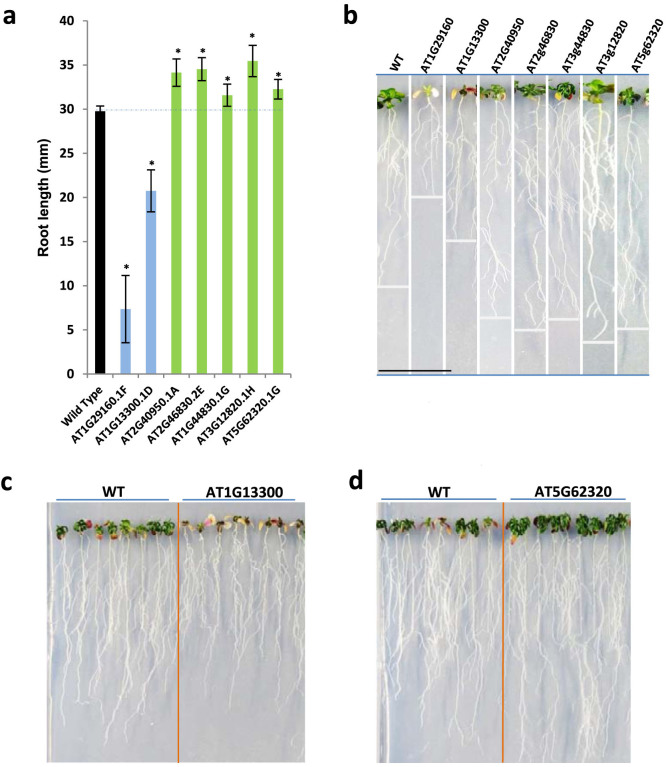
In vivo experimental verification of the predictions. (a) Mean root length corresponding to the wild type (WT, black column) and to 7 other transgenic lines in which a specific gene has been artificially induced. Whiskers represent the standard deviation corresponding to each group. Asterisks denote groups for which the distribution of root lengths is different with respect to the wild type with a 0.01 significance level. (b) Photos of one plant of each of the 8 lines, at the end of the full development process. (c) and (d) Photos of two vertical plates where plants are grown. In both cases, the left (right) photos refer to wild phenotypes (to phenotypes developed by the transgenic line).

**Figure 5:**
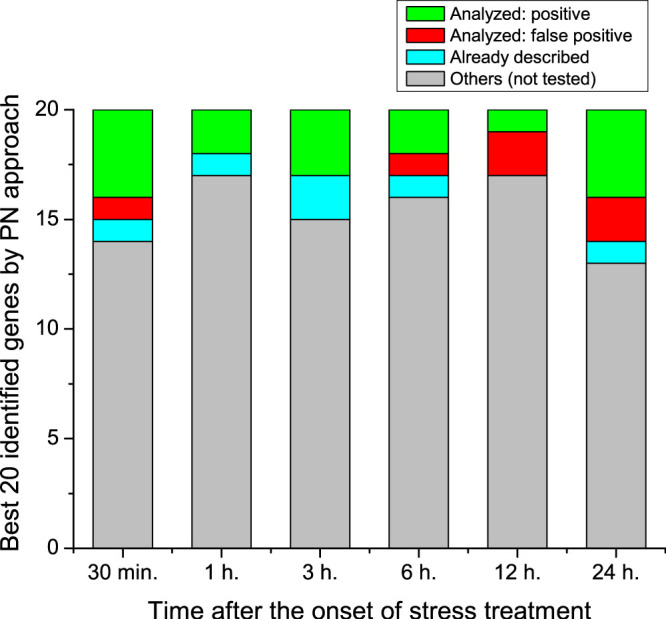
Outcome of the experimental results. Bars account for the 20 most central genes at each time step. For the six time steps considered, bar colors are coded according to the following stipulations: genes previously considered not to be involved in the plant's response to osmotic stress, that were respectively experimentally proven to develop (green) or to fail to develop (red) a statistically significant difference in the phenotype with respect to the wild-type phenotype; (cyan) genes predicted by the parenclitic analysis that were previously associated with the stress response in the Literature; and (gray) previously unknown genes, which could not be tested experimentally, due to their unavailability in the TRANSPLANTA collection.

In order to simplify the problem, here we propose moving from the *n_f_*-dimensional space of features, to the set of *n_f_*(*n_f_* − 1) bidimensional spaces corresponding to pairs of features. For each pair of features *i* and *j*, the values of systems belonging to a given class *c* are used to create a projected constraint 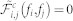, modeling the relationship expected in that plane for systems belonging to that class (see Fig. 1 (b)). While the exact nature of 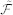 may not be accesible, the aim of this step is the obtention of a reasonable approximation, for instance by means of a polynomial fit, or more generally of a data mining method like Support Vector Machine or Artificial Neural Networks. The selection of the most suitable method will depend on the problem being studied, as for instance on the availability of previous knowledge about relationships between features, or on the number of subjects composing each class.

Once these models have been extracted from available data, we tackle the situation in which a new unlabeled system is made available, and the researcher needs to identify its class by means of a parenclitic network study. This new unlabeled subject will also be described by a vector of *n_f_* features, thus this information can used to calculate the distance between its position in each plane and the corresponding model. Such distance in the *i* − *j* plane is finally used to weight the link between nodes *i* and *j* of the parenclitic network representation - see the red dot and line in Fig. 1 (b) and the resulting topology illustrated in Fig. 1 (c). Notice that in this final step we move from a feature representation (features of all subjects represented in a space) to a subject representation, where one network is constructed for each subject, and nodes represent features.

As discussed in the main text, the resulting network does not only classify the new system as belonging to one of the *n_c_* classes, but can also be used to analyze the characteristics of the system as codified by the network topology, as in the case of the identification of the most central nodes^1^,^2^.

### Representation of Arabidopsis stress response

In this section we present how the parenclitic network reconstruction method can help identifying those genes responsible for the reaction of the *Arabidopsis thaliana* plant to external stresses. The original data set corresponds to the *AtGenExpress project*^17^, including expression levels of 22, 620 genes under 8 different abiotic stresses (*i.e.*, cold, heat, drought, osmotic, salt, wounding and UV-B light) and at six different moments of time (30 min, 1 h, 3 h, 6 h, 12 h and 24 h after the onset of stress treatment). Of these, only the osmotic stress is considered in this work, and the analysis is limited to the *n_f_* = 1, 922 genes composing the transcription factors of Arabidopsis represented in the ATH1 array^18^.

Following the method described in the previous section, the set of systems under analysis is here composed of the status of the plant at a given time step, each one described by a set of features representing the genetic expression of the plant. The objective of the study is the creation of a network representing the genes with an abnormal expression at each time step. In other words, when analyzing data at time *τ*, we create the *n_f_*(*n_f_* − 1) reference models 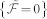 with the data corresponding to all other time steps, and we generate links according to the distance from that reference.

As previously described, during the network reconstruction process it is necessary to define the general form of the reference model 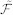. Here, we have chosen the use of a simple linear regression between the expression levels of genes *i* and *j*, such that: 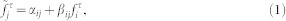

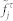
 being the expected value of gene *j* at time *τ*, 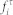 the known expression levels of gene *i*, and *α_ij_* and *β_ij_* two free model parameters. These two coefficients are calculated by means of a linear fit of all values corresponding to other time steps, *i.e.*, minimizing the error of the relation: 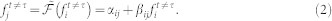
While more complex functions cound have been used for 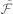, the choice of a linear regression has been motivated by two considerations. First, genetic expression levels are customary transformed in order to have a linear behavior, and the calculation of linear correlations between them is a common procedure in the Literature^6^,^7^,^8^. Second, the reduced number of points available to fit the function 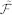 precludes the use of higher-order expressions, as this would result in an overfitting.

Furthermore, the reader should notice that the analysis here presented considers instantaneous interactions between genes, *i.e.* that the value of 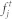 (at time *t*) is only function of 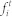, and not of the historical expression of gene *i*. In other words, when the 24 h expression levels are analyzed, we suppose that they are independent on the expression levels at 12 h. While this is clearly a simplification, the low temporal resolution of the available data set prevents a detailed analysis of the delayed influence of gene expressions.

The distance between the expected (corresponding to the model 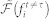) and the real value of gene *j* is then used to weight the link connecting nodes *i* and *j* in the network. More specifically, the weight of the link is the absolute value of the Z-Score of the distance 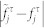.

### Synthetic data analysis

Before being applied to the *Arabidopsis* data set, the proposed methodology has been tested with in-silico generated information, with the additional aim of providing an additional example on how the method works. This synthetic data comprise 10 instances, each one equivalent to the set of expression levels at one time step, and 20 features.

The feature (expression level) of instance *i* at time step *t* is given by the following relation: 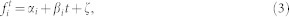
being *α_i_*, *β_i_* and *ζ* random numbers drawn from a normal distribution 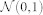. A strong correlation can be found between pairs of features, due to their synchronous evolution with *t*, except of the noise term *ζ* whose objective is to simulate the natural variability observed in genetic expression levels. Finally, features 5 and 10 of instance 10 have been incremented by 2, in order to simulate genetic expression levels that deviate from the normal behavior. The aim of this analysis is then to check whether such abnormal behavior is correctly represented in the resulting parenclitic networks.

### Arabidopsis network analysis

The aim of the analysis is the identification of the most central nodes (i.e., genes) within each of the six parenclitic networks. When a node is strongly central, indeed, it is highly connected, and therefore it is part of a group of many features that deviate pairwise from the expected models.

Due to the characteristics of the network, we have opted for the *α* − *centrality* measure, according to which the centrality of a node is a linear combination of the centralities of those to whom it is connected^19^. If we define a vector 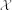 of centralities such that its *i^th^* component *x_i_* is the centrality of the *i*-th node, we have: 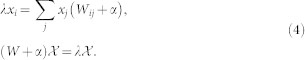
Here, *W* is the weight matrix of the network, and *W_i,j_* codifies the weight of the link connecting nodes *i* and *j*. Notice that this is equivalent to an eigenvalue problem, with constant *α* defining weak connections between all the nodes of the network. In order to have meaningful results, *α* should be smaller than the spectral radius of *W*.

### Osmotic stress tolerance test

For the screening of the transcription factors identified by the parenclitic model, the *Arabidopsis thaliana* inducible lines from Transplanta collection^20^ were used, with the ecotype Columbia (Col-0) as the Wild Type. Each one of the transgenic *Arabidopis* lines of the collection expresses a single *Arabidopsis* transcription factor under the control of the *β*-stradiol inducible promoter.

For osmotic stress screening, seeds from control plants (Col-0) and at least two independent T3 homozygous transgenic lines (Transplanta collection^20^) of each transcription factor were sterilized, vernalized for 2 days at 4°C and plated onto Petri dishes containing 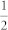 MS medium^21^ supplemented with 10 *μ*M *β*-Stradiol. After 5 days, seedlings were transferred to vertical plates containing 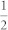 MS medium supplemented with 300 mM Mannitol, 10 *μ*M *β*-stradiol and transferred to a growth chamber at 21°C under long-day growth conditions (16/8 h light/darkness). After 12 days pictures were taken to record the phenotypes, and root elongation measurements were performed with ImageJ software^22^.

## Author Contributions

M.Z. conceived and elaborated the method for parenclitic network reconstruction. J.M.A., J.V.C. and M.G.P. performed the experiment on the Arabidopsis thaliana. M.Z., D.P., P.S., E.M. and S.B. analyzed the data and prepared the figures. M.Z., J.M.A., J.V.C., D.P. and S.B. wrote the text of the Manuscript. All Authors reviewed the Manuscript.

## Figures and Tables

**Table 1:** List of new identified genes involved in osmotic stress responses, revealed by the parenclitic network representation. Gene function was previously unknown in the Literature, and here experimentally proven to develop a statistically significant phenotype in response to osmotic stress. The right most column reports the corresponding *α*-centrality values

Time step	Gene	Name	Centrality
30 m.	AT1G13300	*HRS1*	0.88111
30 m.	AT5G51910	TCP family transcription factor	0.729679
30 m.	AT4G23750	*CRF2*, Cytokinin response factor 2	0.507826
1 h.	AT1G44830	*DREB*	1.0
1 h.	AT3G12820	*MYB10*	0.236686
3 h.	AT2G46830	*CCA1*, Circadian clock associated 1	0.271497
3 h.	AT5G62320	*MYB99*	0.177404
3 h.	AT1G29160	*COG1*	0.148112
6 h.	AT4G16610	C2H2-like zinc finger protein	0.767785
6 h.	AT2G44910	*ATHB-4*	0.689358
12 h.	AT3G61910	*NST2*	0.264721
24 h.	AT1G09540	*MYB61*	0.709785
24 h.	AT2G40950	*BZIP17*	0.551008
24 h.	AT5G62320	*MYB99*	0.482752
24 h.	AT5G04410	*ANAC078*	0.438538
